# Identification of Protein Networks and Biological Pathways Driving the Progression of Atherosclerosis in Human Carotid Arteries Through Mass Spectrometry-Based Proteomics

**DOI:** 10.3390/ijms252413665

**Published:** 2024-12-20

**Authors:** Gergő Kalló, Khadiza Zaman, László Potor, Zoltán Hendrik, Gábor Méhes, Csaba Tóth, Péter Gergely, József Tőzsér, György Balla, József Balla, Laszlo Prokai, Éva Csősz

**Affiliations:** 1 Proteomics Core Facility, Department of Biochemistry and Molecular Biology, Faculty of Medicine, University of Debrecen, 4032 Debrecen, Hungary; kallo.gergo@med.unideb.hu (G.K.); tozser@med.unideb.hu (J.T.); 2 Department of Pharmacology and Neuroscience, University of North Texas Health Science Center, Fort Worth, TX 76107, USA; khadiza.zaman@unthsc.edu; 3 HUN-REN-DE Vascular Pathophysiology Research Group 11003, University of Debrecen, 4032 Debrecen, Hungary; potor.laszlo@med.unideb.hu (L.P.); balla.jozsef@med.unideb.hu (J.B.); 4 Division of Nephrology, Department of Internal Medicine, Faculty of Medicine, University of Debrecen, 4032 Debrecen, Hungary; 5 Department of Forensic Medicine, Faculty of Medicine, University of Debrecen, 4032 Debrecen, Hungary; hendrik.zoltan@med.unideb.hu (Z.H.); gergely.peter@med.unideb.hu (P.G.); 6 Department of Pathology, Faculty of Medicine, University of Debrecen, 4032 Debrecen, Hungary; gabor.mehes@med.unideb.hu; 7 Division of Vascular Surgery, Department of Surgery, Faculty of Medicine, University of Debrecen, 4032 Debrecen, Hungary; toth.csaba@med.unideb.hu; 8 Department of Pediatrics, Faculty of Medicine, University of Debrecen, 4032 Debrecen, Hungary; balla@med.unideb.hu

**Keywords:** atherosclerosis, complicated lesion, human carotid artery, data-dependent LC–MS/MS, data-independent LC–MS/MS, quantitative label-free proteomics, bioinformatics, protein–protein interaction networks, canonical pathways

## Abstract

Vulnerable atherosclerotic plaques, especially hemorrhaged lesions, are the major cause of mortalities related to vascular pathologies. The early identification of vulnerable plaques helps to stratify patients at risk of developing acute vascular events. In this study, proteomics analyses of human carotid artery samples collected from patients with atheromatous plaques and complicated lesions, respectively, as well as from healthy controls were performed. The proteins isolated from the carotid artery samples were analyzed by a bottom-up shotgun approach that relied on nanoflow liquid chromatography–tandem mass spectrometry analyses (LC–MS/MS) using both data-dependent (DDA) and data-independent (DIA) acquisitions. The data obtained by high-resolution DIA analyses displayed a stronger distinction among groups compared to DDA analyses. Differentially expressed proteins were further examined using Ingenuity Pathway Analysis^®^ with focus on pathological and molecular processes driving atherosclerosis. From the more than 150 significantly regulated canonical pathways, atherosclerosis signaling and neutrophil extracellular trap signaling were verified by protein-targeted data extraction. The results of our study are expected to facilitate a better understanding of the disease progression’s molecular drivers and provide inspiration for further multiomics and hypothesis-driven studies.

## 1. Introduction

Vulnerable atherosclerotic plaques, especially hemorrhaged lesions, are the major cause of mortalities linked to vascular diseases [1]. Vulnerable plaques are atherosclerotic lesions that, in the proper milieu, can progress to thrombosis and lead to major adverse cardiovascular events (MACE) and stroke [2,3]. Upon plaque rupture, fibrous cap disruption exposes the thrombogenic contents in the necrotic core causing coronary thrombosis [4]. Early identification of vulnerable plaques helps to stratify patients at risk of developing this acute vascular event. Therefore, extensive research has been conducted to find biomarkers that can enable an early recognition of vulnerable plaques. Studies highlighted that inflammation, oxidative stress, thrombogenesis, and metabolic and epigenetic changes are strongly correlated with plaque vulnerability [5]. Among inflammatory molecules, C-reactive protein, matrix metalloproteases, myeloperoxidase, and cytokines such as IL6 and TNF-α seem to have prognostic value for plaque vulnerability [5,6,7]. Besides protein biomarkers, metabolic changes such as increased serum arginine and cysteine, as well as decreased citrulline concentrations, have also been associated with plaque vulnerability [5,8]. In addition, specific miRNA forms such as miR-122-5p and miR-223-3p were found to be upregulated in patients with unstable plaques [9].

Omics methods have defined new experimental paradigms to understand the pathophysiology of these maladies through systems biology [10]. Genomics, epigenomics, transcriptomics, proteomics, metabolomics, and lipidomics can provide a comprehensive and unbiased view of the molecular changes underlying atherosclerotic plaque biology [9]. Recently, transcriptomic profiling of human carotid endarterectomies by next-generation ribonucleic acid sequencing (RNA-seq) revealed that biological replicates of human complicated lesions and atheromatous plaques clustered together involving many transcripts when compared with healthy tissue segments [11]. To complement previous transcriptomics profiling from the same unique set of human carotid artery samples meeting rigorous classification criteria for inclusion [11], here we report proteomics analyses to identify the differences in the protein expression in atheroma, complicated lesions, and healthy sample groups, which will allow for integrated pathway and network analyses [12].

The classical method of peptide analysis in bottom-up proteomics by liquid chromatography (LC)–tandem mass spectrometry (MS/MS) uses data-dependent acquisition (DDA), which relies on instrument decision algorithms that select precursor ions for MS/MS experiments iteratively based on data extracted from successive survey MS scans [13]. However, major limitations of DDA are the incomplete peptide sampling from complex proteomes [14] with sampled peptides varying among replicate analyses of the same sample [15] and a bias toward covering higher-abundance peptides [16]. Nevertheless, the setup and optimization of the method are well documented [17,18,19], and tools for uncomplicated and reliable data analyses are freely or commercially available [20]. On the other hand, data-independent acquisition (DIA) approaches were introduced to remedy incomplete precursor-ion sampling through collections of MS/MS scans systematically and independently of precursor information [21,22,23,24,25]. Hypothetically, any peptide precursor- and product-ion data within the limit of detection of the instrument can be retrieved from the data files; albeit, the lack of a clear association between precursor ions and their product ions makes data processing challenging and requires software specifically developed for these methods [20,26,27]. DDA generally performs well for detecting high-abundance proteins, but the detection of low-abundance proteins is limited because they may not be selected for MS/MS fragmentation. While it can provide high-quality data for abundant proteins, its ability to detect low-abundance proteins is limited without additional enrichment or complementary techniques [20]. DIA, on the other hand, is more suitable for profiling both high- and low-abundance proteins across the entire dynamic range because it fragments ions within defined windows without relying on their intensity. It offers greater sensitivity, improved quantification of low-abundance proteins, and a wider dynamic range, although it may require more advanced data analysis tools [28]. Overall, hybrid mass spectrometers incorporating high-resolution Orbitrap™ analyzers have been the most frequently used instruments for discovery-driven shotgun proteomics with both DDA [16,17,18,19] and DIA approaches [29,30]. In addition, label-free methods have gained acceptance for quantitative proteomics [31,32]; thus, we selected this approach for DDA- and DIA-based LC–MS/MS analyses of the human carotid endarterectomies that were available for our study.

## 2. Results and Discussion

### 2.1. The DDA-Based Shotgun Proteomics Dataset

In our first approach to processing raw data files, we utilized the freely available MaxQuant platform [33] and searched the Swiss-Prot *Homo sapiens* protein database. Differentially expressed proteins in the analyzed human carotid endarterectomies between atheromatous (A) and healthy vasculature (H), between complicated lesions (C) and H, and between C and A (*n* = 5/group each) were identified by label-free quantification (LFQ) using the MaxLFQ built-in algorithm [34] of the MaxQuant software. Results evaluated by LFQ-Analyst, an interactive web application developed for differential expression analysis and visualization from MaxQuant output files [35], are summarized in Table 1.

A modified data-processing attempt to expand the number of covered proteins using the UniProtKB *Homo sapiens* protein database and still relying on MaxQuant’s algorithm, with the software’s own control of the search’s false discovery rate (FDR ≤ 1%), failed to complete. However, with disabled internal FDR control, the Andromeda search engine of MaxQuant did produce output from this UniprotKB database for follow-up processing by the commercial Scaffold 5 software that applied stringent criteria to satisfy FDR of 1% regarding protein identifications (summarized in Table S1 of the online Supplementary Materials). We were also able to switch to spectral counting for LFQ, which has been found to have higher sensitivity to identify differentially expressed proteins than precursor-ion-based (MS1) quantification such as MaxLFQ in our practice. The spectral counting method of LFQ relies on the number of MS/MS spectra acquired for a certain protein and is, hence, easy to implement and robust [36,37]. This has been the most frequently used method for LFQ in proteomics studies that acquired data by the shotgun method. Our results from this data processing strategy are summarized in Table 2. Quantitative profiles of differentially expressed proteins among the studied groups based on analysis of variance (ANOVA) by Scaffold Quant are listed in Table S2. In addition, Table 2 lists the different post hoc statistical analyses performed with their overall regulation pattern, and the results of ANOVA and the pairwise comparisons were also made available in Table S2. Overall, several findings from processing our DDA analyses were common with those of other studies [38,39], but many more additional and hitherto-not-recognized differences in protein expressions associated with the progression of atherosclerosis have been revealed by our data that we report here.

### 2.2. The DIA-Based Shotgun Proteomics Dataset

DIA has been proposed as a high-throughput, reproducible, and quantitative proteomics technology with high precision that utilizes spectrum reconstruction, sequence or library-based search, and other sequence-independent approaches [21]. However, there are only a few studies conducted on samples from patients with atherosclerosis using DIA-based proteomics. An earlier study identified 4181 proteins from carotid endarterectomies [40], while a more recent study identified 6143 proteins from human carotid plaques [41]. However, there has been no DIA-based differential proteomics study from human carotid endarterectomies to provide insights into atherosclerosis disease progression from atheroma (A) to complicated lesions (C). To analyze our new DIA dataset addressing this information gap, we employed Spectronaut (version 18, with its Pulsar search identification mode). Like upon handling the DDA dataset with the combined Andromeda–Scaffold 5 procedure whose results were summarized in Table 2, we relied on the UniProtKB *Homo sapiens* protein database to search the acquired raw files. Detailed results of statistical analyses (ANOVA and pairwise comparisons between the study groups by post hoc *t*-tests) were collected in Table S3.

**Figure 1:**
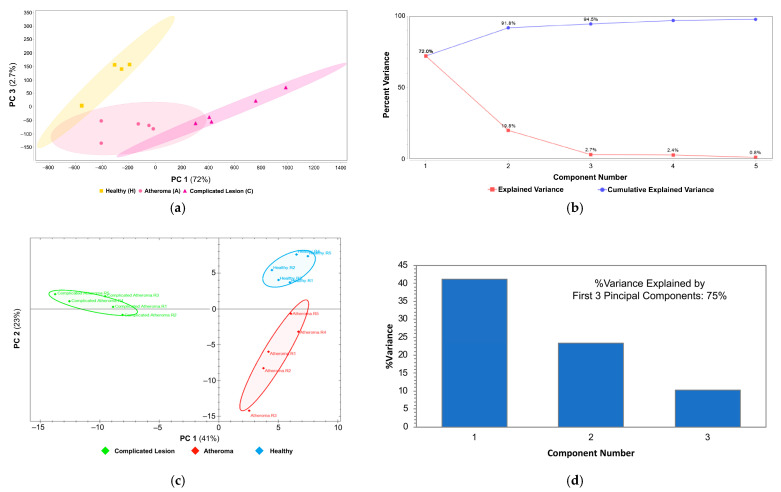
Similarities among the classified human carotid endarterectomies with DDA and DIA methods of label-free proteomics. (**a**) PCA plot constructed by Scaffold Quant for DDA-based analysis showing similarities among the healthy (H, yellow box), atheroma (A, pink circle), and complicated lesion (C, purple triangle) groups; (**b**) scree plot explaining the variance by each principal component (PC) from Scaffold Quant results; (**c**) PCA plot constructed by Spectronaut showing similarities among the healthy (H, blue), atheroma (A, red), and complicated lesion (C, green) groups from DIA-based analysis; (**d**) components bar plot: how the first three PCs explain the variance from the Spectronaut results.

Overall, DIA-based proteomics resulted in the identification of close to 4000 proteins at a 1% FDR. However, the total numbers of differentially regulated proteins [42] were similar to our DDA method, as shown in Table 3. A comprehensive graphical representation of the similarities and dissimilarities in protein expression profiles from the DDA- and DIA-based proteomics pipeline is displayed in Figure 1 by principal component analysis (PCA) plots showing sample clustering constructed from the protein profiles [43]. The DDA proteomics results show similarities among the sample groups; samples from the H and A groups are closer; in contrast, the samples from the C group are more divergent from the H group than from A. Overall, data obtained by DIA-based analyses display a stronger distinction among groups (Figure 1c) compared to the DDA analysis.

### 2.3. Comparative and Comprehensive Analyses of the DDA and DIA Results by Ingenuity Pathway Analysis^®^

About one third of the proteins identified from our DDA dataset and characteristic to complicated lesions (C, see Table S1) were also identified in a mouse study examining stable and unstable lesions, as well as in human samples [38,39]. In addition, many proteins belonged to the antimicrobial and immunomodulatory peptide family according to the UDAMP Unified Human Antimicrobial and Immunomodulatory Peptide Database [44]. These proteins can be indicators of innate immune system activation having roles not only in antimicrobial defense but in protection against oxidative damage. This protection can be useful in complicated lesions where the harmful effects of heme dominate.

Among the proteins whose level increased specifically in complicated lesions, β2 microglobulin (B2M) and the proteins S100A8 and S100A9 were earlier identified as risk factors for cardiovascular events [45,46]. Their levels were significantly increased in the C sample group indicating the strong link between these proteins and complicated lesions. However, though S100A8 and S100A9 were identified in both DDA and DIA datasets, we did not find B2M to be a significantly regulated protein in the DIA dataset. Although our data did not give further evidence of whether these observed changes are causes or consequences, they might be part of a defense mechanism in response to bleeding-induced heme stress considering the protective properties of these proteins.

Beyond focusing on these specific findings and comparisons of the number of proteins found to be affected by atherosclerosis based on the results summarized in Table 2 and Table 3, follow-up bioinformatics-centered analyses were conducted to gain insights into the protein–protein interaction networks and biological pathways driving the progression of atherosclerosis to vulnerable plaques. Figure S1 displays a comparison of the DDA and DIA approaches considering proteins significantly affected by the disease and also mapped by Ingenuity Pathway Analysis^®^ (IPA^®^). Overall, the two data acquisition strategies yielded surprisingly complementary information. Therefore, we performed IPA^®^ on the combined results of the DDA and DIA datasets. The differentially regulated proteins from the combined DDA and DIA datasets were submitted to IPA^®^ to derive bioinformatics annotations along with relevant protein–protein interaction networks, as well as associated biological functions and processes. IPA^®^’s overlay function was used to display and compare proteome profiles relying on pairwise comparisons in the pathways and networks not only between the biological categories but also dataset overlays of the results obtained through the two acquisition methods (i.e., DDA and DIA) that relied on the same instrument and had closely matched conditions in the analyses, as well as the same tissue samples.

**Figure 2:**
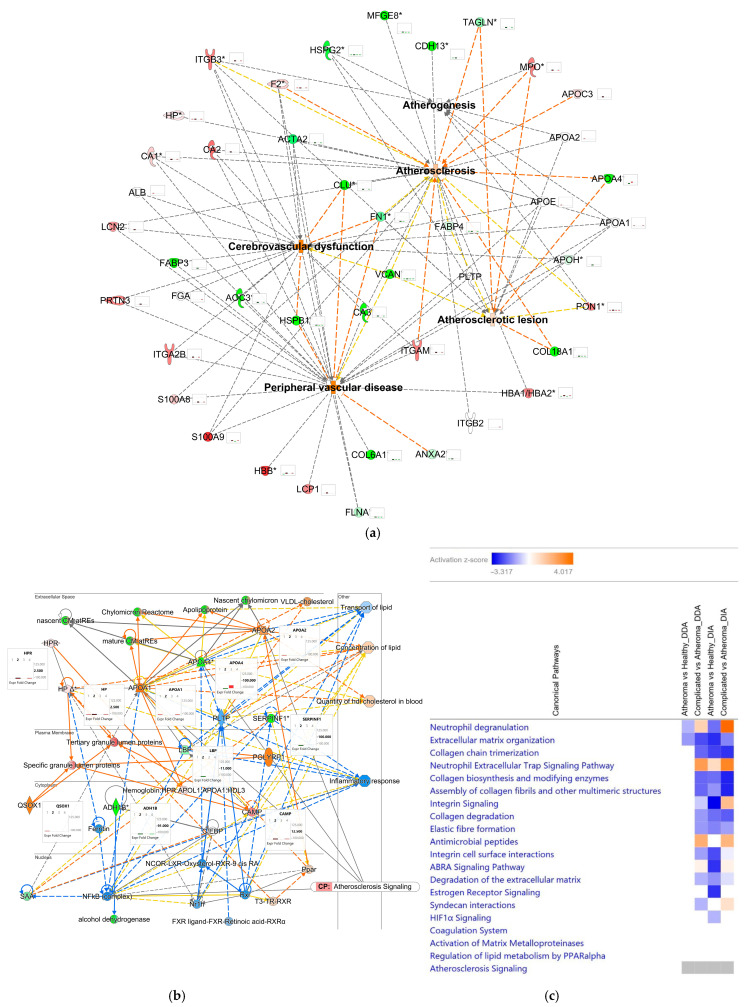
IPA^®^ mapping of proteins regulated in different forms of atherosclerotic lesions obtained from human carotid arteries. (**a**) The protein–protein interaction network of proteins related to atherogenesis, atherosclerosis, atherosclerotic lesions, cerebrovascular dysfunction, and peripheral vascular disease. Molecule activation predictor (MAP) showing the overall effect of complicated atherosclerotic lesions: blue dashed line—inhibition/decrease; orange dashed line—activation/increase; yellow dashed line—cannot be predicted; orange solid line—activation; blue solid line—inhibition. Insets: 1st bar A versus C, DDA; 2nd bar—C versus H, DDA; 3rd bar—A versus H, DIA; 4th bar—C versus H, DIA. (**b**) An IPA^®^ protein interaction network linked to cardiac dysfunction, lipid metabolism, and small-molecule biochemistry. Insets: 1st bar A versus C, DDA; 2nd bar—C versus H, DDA; 3rd bar—A versus H, DIA; 4th bar—C versus H, DIA. Map shows the overall effect of complicated atherosclerotic lesions (C samples). CP—canonical pathway; red—upregulation; green—downregulation; shade of color is indicative of the extent of change in expression; solid line—direct relationship; dashed line—indirect relationship; yellow dashed or solid line—activity cannot be predicted. Abbreviation of proteins are listed in Table S8. Asterisks indicate multiple protein isoforms from the same gene. (**c**) Canonical pathway comparison analysis: 1st panel—A versus H, DDA; 2nd panel—C versus A, DDA; 3rd panel—A versus H, DIA; 4th panel—C versus A, DIA. Blue box and orange box designate inhibition/decrease and activation/increase of the pathway, respectively, with z-score indicated by shade of color (scale on the top, with white stipulating no activation). Grey box indicates that IPA^®^ could not make a prediction.

Proteins involved in selected aspects of the studied conditions including atherogenesis, atherosclerosis, atherosclerotic lesion, peripheral vascular disease, and cerebrovascular dysfunction, which is a common aftermath of atherosclerotic events [47,48,49,50], are highlighted by the networks shown in Figure 2a,b. We may state that, in most of the cases, the type of data acquisition did not impact the direction of change; however, DIA seemed slightly more sensitive compared to DDA based on the z-scores assigned by IPA^®^ to the affected top canonical pathways (Figure 2c).

We could also observe the overall functional changes characteristic of atherosclerosis, indicating the buildup of lipids and a decrease in lipid transportation and inflammatory response, culminating in atherosclerotic lesions. This representation not only helps to visualize the level of congruency between different methods but also enables screening for potential preclinical endpoints of the disease. For example, clusterin (CLU, which is a molecular player in atherosclerosis signaling [51] and a node connecting atherosclerosis with cerebrovascular dysfunction in our network displayed in Figure 2a) was shown to be downregulated in complicated lesion (C) samples by both the DDA and DIA results.

Altogether, our IPA^®^-based analysis also revealed more than 150 canonical pathways to be significantly regulated (Table S6). Out of the top canonical pathways (Figure 2c), we focused on verifying two atherosclerosis-related canonical pathways by a targeted approach from our DIA dataset.

### 2.4. Examination of the Complex Landscape of Complicated Atherosclerotic Lesions and Identification of Potential Biomarkers from Selected Canonical Pathways

**Figure 3:**
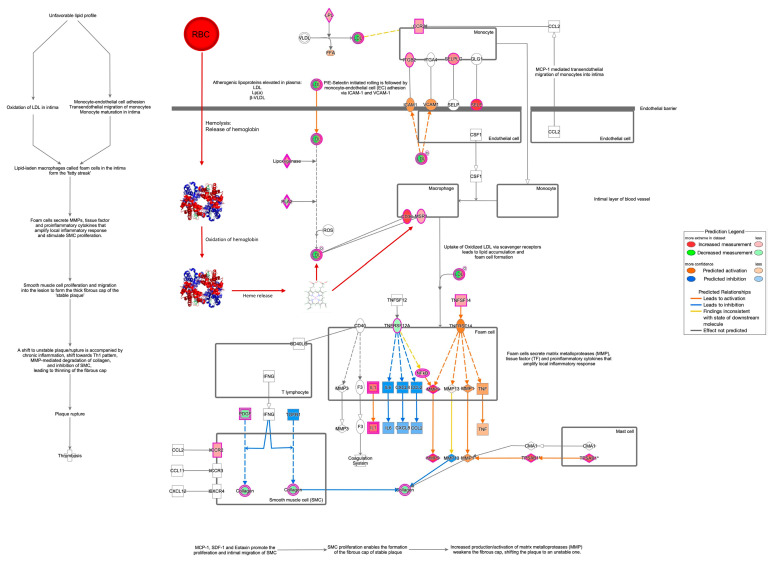
IPA^®^’s atherosclerosis signaling canonical pathway complemented with previous findings by our group regarding the role of red blood cell lysis followed by hemoglobin release, hemoglobin oxidation, and heme release [20,21,22]. Symbols with purple borders indicate proteins in the pathway that showed statistically significant differences in expression among the study groups (Table S2). Asterisks indicate that multiple protein identifiers (isoforms) in the input file were mapped to the same gene. The meaning of colors for shapes and lines is shown in the inset. Complemented steps are marked with red arrows.

To prevent life threatening events associated with atherogenesis, unravelling early biomarkers and their involvement in atherosclerotic mechanisms is of prime importance. However, issues such as sample and tissue availability, plaque complexities, and absence of proper control often cloud assessments [36]. Considering that DIA analysis has become a robust mass spectrometric method for potentially all-inclusive targeted protein quantitation [52,53,54], we performed a reanalysis of our DIA-based proteomics dataset to verify selected results implicated by IPA^®^ (i.e., without additional data acquisition). To this end, the raw data files were processed by using Scaffold DIA (version 3.2.1). This software enabled targeted quantification through an approach that resembled parallel reaction monitoring (PRM) even when the DIA dataset was obtained in an untargeted manner. Overall, we found 132 targets quantified without false discovery. These proteins, together with lists of transitions used for their quantitation, are listed in Table S7. The value of this approach is demonstrated below through the evaluation of two canonical pathways we chose as examples: atherosclerosis signaling (log *p* = 11.6, Figure 3) and the neutrophil extracellular trap (NET) signaling (log *p* = 12.7, Figure S2) with a focus on comparisons of endarterectomized carotid arteries with complicated lesions with those with non-hemorrhaged plaques.

**Figure 4:**
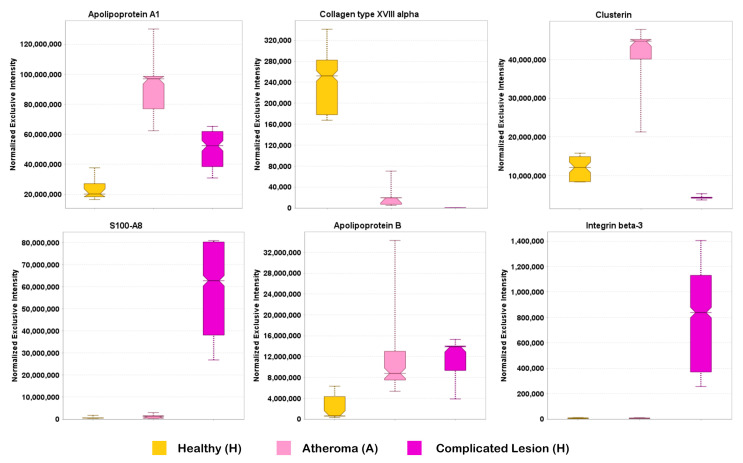
Quantitative survey by the Scaffold DIA processing methods measuring the differential expression of key proteins in IPA^®^’s atherosclerosis signaling canonical pathway. Box plots generated by the software: H (yellow boxes), A (pink boxes), and C samples (purple boxes). ANOVA followed by post hoc Tukey–Kramer tests (*n* = 5, *p* < 0.05): statistically significant difference between A and H for apolipoprotein A1, collagen type XVIII alpha, and clusterin; statistically significant difference between C and H for apolipoprotein A1, collagen type XVIII alpha, clusterin, S100A8, apolipoprotein B, and integrin beta-3; statistically significant difference between C and A for apolipoprotein A1, collagen type XVIII alpha, clusterin, S100A8, and integrin beta-3.

Specifically, IPA^®^ could not make predictions about inhibition or activation of the atherosclerosis signaling canonical pathway from our results. However, the analyses revealed the impact of oxidized-low-density-lipoproteins (oxLDLs) on macrophages through the upregulation of CD36, a scavenger receptor involved in immunity, metabolism, and angiogenesis, as well as through the macrophage scavenger receptor 1 (MSR1) [55]. This pathway can be considered downstream of the steps elucidated earlier by our group [56,57,58]. Accordingly, red blood cells in the ruptured plaques are lysed and the released hemoglobin is oxidized, forming ferrylhemoglobin and methemoglobin, followed by the dissociation of the hemoglobin polypeptide chains and heme [58]. Heme enhances the oxidation of LDL [59,60] and activates macrophages leading to the deposition of free and esterified cholesterol, as well as to the generation of foam cells inducing the secretion of matrix metalloproteases (MPPs), tissue factors, and proinflammatory cytokines that amplify the local inflammatory response [59]. Overall, foam cell formation is associated with a shift in plaque progression to an unstable stage [60]. With Scaffold DIA’s targeted data extraction method (Figure 4), we were also able to verify the involvement of several proteins in the atherosclerosis signaling canonical pathway of IPA^®^.

Apolipoproteins (APOs) are parts of lipoproteins such as the low-density lipoprotein (LDL, Figure 3) and the high-density lipoprotein (HDL). Elevated LDL is a known risk factor of coronary atherosclerosis [61]. However, the ratio of APOB/APOA1 can also provide indication of early, subclinical atherosclerosis [62], and a higher ratio reflects the severity of atherosclerosis, yet the exact mechanism of how this ratio plays a role in this regard is not well understood. While APOA1 is known to have protective effects, APOB is mostly involved in facilitating atherosclerosis [63,64]. A study conducted by optical coherence tomography (OCT) showed an association between the APOB/APOA1 ratio and atherosclerosis plaques [65]. The OCT characteristics revealed that the APOB/APOA1 ratio was higher in patients with severe calcification, erosion, plaque rupture, and thrombus. Overall, APOB/APOA1 has a better predictive value for atherosclerotic plaques compared to other biomarkers like LDL-C. However, due to the inherent problems in extracting them from the extracellular lipid-rich matrixes, diagnostic methods rely heavily upon genotyping, imaging, and immunosorbent assays which are time-consuming and not cost-effective. Though there have been several reports on lipoproteomics [66], sample heterogeneity, complexities in lipoproteome purification, and varying mass spectrometry analysis performance have resulted in differences from study to study [67]. To our knowledge, this is the first DIA-based targeted proteomics verification of a high APOB/APOA1 ratio found in complicated atheroma lesions, as shown in Figure 3 and Figure 4, inferring a higher occurrence of erosion, rupture, and calcification. This finding could pave the way to establish APOB/APOA1 as a potential early diagnostic tool.

Collagens are extracellular matrix (ECM) proteins that play vital roles during calcification and plaque formation [68]. Plaque formation was found to be a healing response to endothelial injury, with collagen synthesis being a major factor [69]. Under circumstances of insufficient collagens, plaque calcification may occur. Thus, the lack of collagen has been indicative of a more pathogenic phenomenon. Collagen tripeptides have been shown to reduce plaques and have been beneficial against aortic plaque development [70,71]. Thus, our analyses verified a downregulated collagen profile in normal atheroma (A) and complete absence of collagens in complicated lesions (C) when compared to the healthy individuals (H) proving both its predictive and potential therapeutic value. Integrins can also interact with ECM proteins, and plaque stability is highly dependent on their expression levels according to a study [54] that reported an upregulation pattern of integrins. Our focused processing of the DIA raw data files also validated this observation by showing significant upregulation of integrin β-3 (Figure 4). In this context, strategies have also been developed to block integrin α|β in platelets as therapeutic interventions against the progression of normal atheroma (A) and complete absence of collagens in complicated lesions (C) [72,73].

Clusterins (CLUs) mainly function as extracellular chaperones averting aggregation of non-native proteins. The role of CLU in atheroma is poorly understood, but few studies have shown the upregulated pattern of CLU as the disease progresses [74]. In this investigation, though CLU was upregulated in the normal atheroma lesions, its amount was very low in complicated lesions.

S100A proteins are neutrophil-derived proteins that characterize the inflammation signature in atherosclerotic plaques [75]. S100A8, belonging to the S100 calgranulin family, can be associated with the degree of carotid atherosclerosis [76]. Elevated levels of S100A8 in plasma samples have also been linked with an increased risk of future cardiovascular dysfunction [76]. Thus, S100A8 can be established as a valuable biomarker and therapeutic component in cardiovascular dysfunctions like atheroma. In this study, in line with previous findings, we confirmed high expression levels of S100A8 in complicated lesions, possibly by recruiting and activating neutrophils and monocytes in arterial walls [76,77,78].

**Figure 5:**
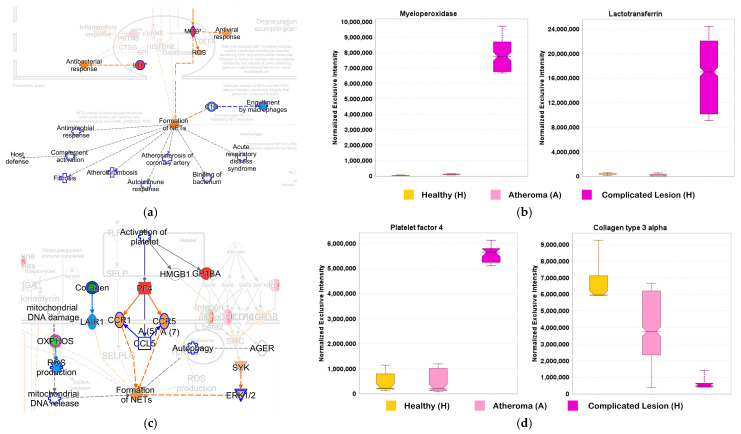
A Scaffold DIA-based quantitative survey of proteins identified as surrogate endpoints involved in the neutrophil extracellular trap (NET) signaling canonical pathway shown fully in Figure S2. (**a**) View focused on myeloperoxidase (MPO) and lactotransferrin (LTF) with the Path Tracer of IPA^®^; (**b**) box plots generated by Scaffold DIA for LTF and MPO from H (yellow boxes), A (pink boxes), and C samples (purple boxes); ANOVA followed by post hoc Tukey–Kramer tests (*n* = 5, *p* < 0.05): statistically significant difference between C and H, as well as C and A, for both LTF and MPO; (**c**) view focused on platelet factor 4 (PF4) and collagen with the Path Tracer of IPA^®^ and (**d**) box plots generated by Scaffold DIA for collagen type 3 alpha and PF4 from H (yellow boxes), A (pink boxes), and C samples (purple boxes); ANOVA followed by post hoc Tukey–Kramer tests (*n* = 5, *p* < 0.05): statistically significant difference between C and H for both collagen type 3 alpha and PF4; statistically significant difference between C and A for PF4. In figures (**a**,**c**), blue indicates a decrease and inhibition, while red and orange denote an increase and activation, respectively.

Atherosclerosis involves a variety of blood and immune cells. Among these cells, neutrophils are known to be a vital defense mechanism against microbial invasion by forming so-called neutrophil extracellular traps (NETs) [79,80]. These traps are web-like structures formed by activated neutrophils and can be considered as an amalgamation of histones, decondensed chromatin, nuclear and cellular proteins, proteases, and azurophilic granules along with holding fibrin and coagulation factors from the circulatory system. This interaction and crosslinking of structures are considered a major pathogenic route for atherosclerotic plaque formation [81]. Thus, owing to their mechanistic origin, proteins involved in this pathway can be used as therapeutic agents to decelerate atherosclerosis progression. IPA^®^ indeed revealed NET signaling as one of the most highly regulated pathways in complicated lesions. However, by using the comparison analysis tool in IPA^®^, the extent of activation was more intense in the case of complicated lesions (Figure 2c). Owing to the mechanistic significance of this pathway in atherosclerosis pathophysiology, we verified proteins involved in the NET signaling pathway through data extraction using Scaffold DIA (Figure 5).

Among these proteins, myeloperoxidase (MPO) and lactotransferrin (LTF) were seen to be directly involved in the formation of NETs. MPO, which was upregulated in complicated plaques (C) according to our results (Figure 5a,b), is a known biomarker for cardiovascular diseases where high levels are usually associated with an elevated risk of developing coronary artery disease [81]. Among the many roles of MPO, it is part of the innate immune response and has potent antimicrobial properties [82] and is instrumental in forming atherosclerotic lesions, primarily by oxidizing low-density lipoproteins [83]. Owing to the numerous roles of MPO in atheroma-proteomics-based validation studies can help to establish it as a potential therapeutic biomarker [84]. LTF is also a neutrophil-derived substance and is commonly known as iron-binding glycoprotein and has antibacterial, anti-inflammatory, and antioxidant properties [85]. In response to atherosclerosis, neutrophils release more LTF. Higher circulating levels of LTF corresponded to a higher risk of atherosclerosis-related ischemic heart disease [86]. LTF also showed high affinity towards MPO-modified lipoproteins (Mox-LDLs) [87]. These Mox-LDLs reside in macrophages forming foam cells which are prognostic characteristics of atherosclerosis. LTF was responsible for inhibiting the accumulation of cholesterol in these types of macrophages and thus proving its potency as a potential therapeutic agent. With our study, we validated high levels of MPO and LTF in the complicated atheroma group (Figure 5a,b), further emphasizing the associated pathological processes driving the progression of atherosclerosis.

Platelets are primarily involved in hemostasis; however, recent studies have established their roles in immunological, cardiovascular, and other pathologies [73]. In the context of atherosclerosis, platelets are mainly involved in thrombus formation that follows the rupture of a plaque [88]. Platelets can also impact the firmness of the plaque by controlling the microenvironment of the plaque core. In our targeted investigation, we verified a significant upregulation of platelet factor 4 (PF4, Figure 5c,d) in complicated lesions. Platelet factors are released by platelets and help to recruit and activate other cells [89], which can lead to inflammatory processes observed during atherogenesis. Current therapeutic strategies have implicated blockers of platelet activation [90]. However, since the involvement of platelets is a recent observation, more studies are required to establish it as a potential therapeutic target.

Collagens are one of the most abundant proteins in the human body and are high-affinity ligands for the broadly expressed leukocyte-associated–immunoglobulin-like receptor-1 (LAIR-1) [91]. LAIR-1 is known for the inhibition of neutrophil activation and NET formation [92,93]. Our DIA investigation revealed the downregulation of collagen type 3 alpha in complicated lesions (Figure 5c,d); therefore, LAIR-1 is not able to inhibit the neutrophil activation and NET formation based on our results.

### 2.5. Comparative Analysis of Transcriptomic and Proteomic Datasets

Our research group previously reported the transcriptomic profiling of human carotid endarterectomies by next-generation ribonucleic acid sequencing (RNA-seq) [11]. The healthy vasculature, atheromatous, and complicated lesion samples analyzed by RNA-seq were used in our study to explore the proteome-level changes. Overall, the affected genes according to RNA-seq analyses were mainly related to macrophage and neutrophil activation, angiogenesis, and iron transport [11]. IPA^®^ from the results of our proteomics study also revealed pathways related to neutrophil granulocyte and macrophage activation, as well as iron transport and homeostasis (Table S6). However, many other pathways were found to be significantly affected upon progression of atheromatous plaques towards complicated lesions. Our Table S9 summarized the transcriptome-versus-proteome profile regarding atherosclerosis signaling and NET signaling, which were shared IPA^®^ canonical pathways significantly affected based on the results of both omics methods. These summaries reinforced that, while transcriptomics generally offered a greater depth compared to proteomics considering the number of molecules detected, proteome profiling may have unique rewards and may also outperform transcriptome profiling regarding interpretable outcomes of omics-based human disease studies [92].

Neutrophil and macrophage activation in atherosclerosis plays a critical role in the progression of the disease, as confirmed both by previous transcriptomics [11] and the proteomics profiling reported here. Both cell types contribute to inflammation, plaque formation, and plaque destabilization, leading to potential cardiovascular events such as heart attack (a MACE) or stroke [93]. Neutrophils are the first responders to inflammation. In atherosclerosis, they are recruited to the site of the plaque by pro-inflammatory signals secreted by endothelial cells, smooth muscle cells, and macrophages [93]. Neutrophils can undergo NETosis, releasing NET as described above. Activated macrophages, particularly those with the M1 phenotype, secrete cytokines and chemokines that promote inflammation and the recruitment of additional immune cells. M1 macrophages are also involved in the uptake of oxidized-low-density lipoproteins (oxLDLs) through scavenger receptors, which leads to foam cell formation, a hallmark of atherosclerotic plaques [94]. Macrophages contribute to plaque destabilization by secreting proteases (such as matrix metalloproteinases), which degrade the fibrous cap of the plaque, making it more prone to rupture. Plaque rupture can trigger thrombus formation, leading to acute cardiovascular events. Crosstalk between neutrophils and macrophages is also crucial in atherosclerosis. Both cell types produce cytokines and chemokines that reinforce each other’s activation, e.g., neutrophils can release IL-17, which promotes macrophage activation and enhances the inflammatory response [95]. Additionally, both neutrophils and macrophages contribute to the formation of foam cells, which accelerate plaque development.

In atherosclerosis, iron can accumulate in various components of the plaque, including foam cells. High concentrations of iron in macrophages and other cells within the plaque can exacerbate inflammation and tissue damage. Macrophages in the plaque can take up and store excess iron through ferritin or release it through ferroportin. When macrophages undergo necrosis or apoptosis, iron is released into the plaque environment, contributing to further oxidative stress and plaque instability [96]. Iron in the form of ferritin or heme from dying cells within plaques may contribute to the degradation of the fibrous cap by promoting matrix metalloproteinase activity. This degradation weakens the plaque structure, increasing the risk of plaque rupture and thrombosis, which can lead to heart attacks or strokes [97].

Additionally, several additional pathways identified by RNA-Seq were also identified in our study such as apoptosis, complement system activation, wound healing, endocytosis, and calcium signaling and transport (Table S6), highlighting the importance of these biological pathways in the development of atherosclerotic plaques.

In conclusion, many potential associations besides the discussed atherosclerosis signaling and NET signaling canonical pathways but eventually captured by the presented proteomics datasets based on high-resolution mass spectrometry (Figure 2c) remain to be explored. Together with the available transcriptome profiles [11], we anticipate that information about proteome profiles of classified atherosclerotic human carotid endarterectomies from our datasets will inspire multiomics data analyses [12,98] and also prompt hypothesis-driven experimental studies that contribute to a better understanding of the associated pathophysiology and to the improvement of disease prognosis. Moreover, emerging ion mobility technologies, such as trapped ion mobility spectrometry (TIMS) or parallel accumulation serial fragmentation (PASEF) [99], can add further depth to the analysis of atherosclerotic plaques in future proteomics studies focusing on atherosclerosis. The application of these techniques may help differentiate proteins that are differentially abundant across various plaque regions, such as in the fibrous cap versus the lipid core and can be used to study proteins in specific cell types (e.g., endothelial cells, smooth muscle cells, and macrophages) within plaques, enabling the exploration of cell-specific protein distributions. TIMS and PASEF also allow for the detailed investigation of disease-associated modifications such as glycosylation or phosphorylation in low-abundance proteins. For potential clinical translation, findings of discovery-driven studies should be validated on a larger cohort in separate follow-up studies relying on targeted proteomics nevertheless [52,100].

## 3. Materials and Methods

### 3.1. Study Approval and Sample Collection

Study approval, as well as collection and selection of study specimens, were described in our previous publication [11]. The collection of carotid arteries was approved by the Scientific and Research Ethics Committee of the Scientific Council of Health of the Hungarian Government under the registration number of DE OEC RKEB/IKEB 3712-2012. Written informed consent was received from the participants. Healthy carotid arteries for controls were obtained from cadavers of suicide or traumatic events without cardiovascular diseases from the Department of Forensic Medicine (Regional Research Ethical Committee, Project No. 5038-2018). Briefly, atherosclerotic samples were obtained from patients by carotid endarterectomies, and healthy carotid arteries were harvested from cadavers of suicide or fatal trauma victims without cardiovascular diseases. All samples were classified by a pathologist according to guidelines by the American Heart Association [101] as atheromatous (A) and complicated lesions (C) and were identical with those having met specified study criteria and included in high-throughput global transcriptomic analyses by RNA-seq as reported earlier [11]. Specifically, inclusion criteria were as follows: samples received within 1 h after endarterectomy, no RNA and protein degradation occurred, blood clot in the artery was absent, and collection of samples was conducted appropriately.

### 3.2. Sample Preparation for Shotgun Proteomics

Carotid artery samples (N = 5 per study group) were snap frozen and kept at −80 °C till the analyses. The samples were homogenized in liquid nitrogen and dissolved in protein lysis buffer (10 mM Tris-HCl, 5 mM EDTA, 150 mM NaCl (pH 7.2), 1% Triton X-100, 0.5% Nonidet P-40, and protease inhibitors (Complete Mini; F. Hoffmann-La Roche Ltd., Basel, Switzerland)). The supernatant was collected, and protein content was measured using a commercial dye-binding method (microBCA; Bio-Rad, Hercules, CA, USA), and an aliquot containing 40 µg protein was subjected to in-solution trypsin digestion. Proteins were denatured with 6 M urea (Bio-Rad, Hercules, CA, USA) for 30 min; thereafter, they were reduced with 10 mM dithiothreitol (Bio-Rad) at 37 °C for 60 min and further alkylated with 20 mM iodoacetamide (Bio-Rad) in the dark for 45 min. Before trypsin digestion, samples were diluted with 25 mM ammonium bicarbonate (Sigma-Aldrich, St. Louis, MO, USA) to decrease the urea concentration to 1 M. Digestion was realized at 37 °C overnight by adding MS-grade modified trypsin (Sciex, Framingham, MA, USA) in a 1:25 enzyme-to-protein ratio. The digested proteins were dried in speed-vac and dissolved in 1% aqueous formic acid solution. The samples were desalted with C18 PierceTip (Thermo Fisher Scientific, San Jose, CA, USA), dried, and re-dissolved in 50 µL 1% aqueous formic acid solution before analyses.

### 3.3. LC–MS/MS Analyses

The digested samples were analyzed first using DDA-based LC–ESI-MS/MS on an Orbitrap Fusion™ Tribrid™ mass spectrometer connected online to an EASY nLC-1200 system (both from Thermo Fisher Scientific, San Jose, CA, USA). Five µL aliquots of the digested samples were injected at a constant flow of 1 mL/min to a 2 cm × 75 μm i.d. Acclaim™ trap column packed with 3 µm PepMap™ 100 C18 particles (Thermo Fisher Scientific). Then, analytical nanoflow separations were realized using a 15 cm × 75 μm i.d. Acclaim™ column also packed with 2 µm PepMap™ 100 C18 particles (Thermo Fisher Scientific) with elution at a 300 nL/min flow rate with a 2.5 h gradient. The chromatographic separation was performed by using a gradient of 5–7% solvent B over 5 min, followed by a rise to 15% of solvent B over 50 min, and then to 35% solvent B over 60 min. Thereafter, solvent B was increased to 40% over 28 min and then to 85% over 5 min, followed by a 10 min rise to 85% of solvent B, after which the system returned to 5% solvent B in 1 min for a 16 min hold-on. The nanoelectrospray ion source (Nanospray Flex™, Thermo Fisher Scientific) was operated at 2.3 kV in the positive-ion mode, and the ion-transfer tube temperature was 275 °C. For data-dependent acquisition (DDA), full-scan mass spectra (MS) were acquired from *m*/*z* of 350 to 1600 at a mass resolution set to 60,000 at an *m*/*z* of 200 in the Orbitrap with an automatic gain control (AGC) target of 40,000, and up to 14 MS-dependent MS/MS were obtained using collision-induced dissociation (CID) with helium as the collision gas in the ion trap with an AGC target of 2000. Each MS/MS spectrum was acquired with multiply charged peptide ions (z ≥ 2) at a 35% relative collision energy. After selection of the precursor ion to be fragmented, 45 s of dynamic exclusion was applied.

For DIA, the samples, nano-LC separations, and ESI settings were the same as described above for the DDA analyses. Full-scan mass spectra were acquired from an *m*/*z* of 385 to 1015 at mass resolution set to 120,000 at *m*/*z* 200 in the Orbitrap and with automatic gain control (AGC) target of 400,000. DIA scans were acquired by using 24 *m*/*z* isolation windows in the 385 to 1015 *m*/*z* precursor range, and fragmentation was performed using HCD using a 33% normalized collision energy and detecting the product ions in the Orbitrap™ analyzer with an AGC target of 50,000.

### 3.4. Data Analysis

#### 3.4.1. DDA-Based Shotgun Proteomics

Raw data files acquired by DDA were searched using MaxQuant (versions 2.4.9.0 and 2.6.4.0; https://maxquant.org accessed on 20 February 2024 and 3 September 2024, respectively). A parent-ion mass tolerance of 4.5 ppm, fragment-ion mass tolerance of 0.50 Da, and one missed cleavage were set as the search filters. Fixed modifications included carbamidomethylation of cysteine, while methionine oxidation and trioxidation of cysteine were chosen as variable modifications. First, we searched MS/MS spectra against the Swiss-Prot *Homo sapiens* protein sequence database (20,346 entries) with MaxQuant’s false discovery control enabled (PSM FDR and protein FDR set to 0.01). The site decoy fraction was 0.01, and the minimum peptide ratio for MaxLFQ was 1. Differentially expresses proteins between sample categories were obtained by submitting the search result to LFQ-Analyst [35]. Missing-value imputation was realized with MinDet, and the program’s default Benjamini–Hochberg FDR correction was selected.

In another workflow to process the DDA raw data files, MaxQuant was used with its FDR control disabled (i.e., PSM FDR and protein FDR were set to 1.00), and MS/MS spectra were searched against the UniProtKB protein sequence database (species: *Homo sapiens*, 2023; 204,500 entries). The site decoy fraction and minimum peptide ratio for MaxLFQ remained at 0.01 and 1, respectively. Search results were validated by Scaffold (version 5.3.3; Proteome Software, Portland, OR, USA) to meet the criteria of peptide and protein identifications at a 1% FDR by MSFragger [102], Peptide Prophet [103], and Protein Prophet [104] algorithms, and at least two identified unique peptides were considered for subsequent LFQ by spectral counting [105]. Differentially regulated proteins in the dataset were obtained by one-way ANOVA with *p* < 0.05 considered significant based on spectral counts. Then, pairwise comparisons between groups involving these proteins relied on *t*-tests [106]. Missing values, if any, were handled using Scaffold’s default method and settings. PCA plots were obtained using Scaffold Quant (version 5.03; Proteome Software, Portland, OR, USA).

#### 3.4.2. DIA-Based Shotgun Proteomics

To analyze the DIA dataset, we employed Spectronaut (version 18) with the UniProtKB protein database. Spectronaut uses peak properties and a target decoy approach to identify peptides. The Pulsar search identification mode with directDIA + (Deep) workflow was used with the PSM FDR, peptide FDR, and protein group FDR set to 0.01 (1%). A machine learning scheme is used to determine a score for the peptide precursors and decoys. Then, the target and decoy distributions are fitted using a kernel-density approach [107]. These distributions are used to calculate the estimation of the run-wise FDR and posterior error probability (PEP). Spectronaut’s intensity-based fragment ion selection strategy was applied for LFQ of the identified proteins, and statistically significant differences in protein expressions were obtained with the program’s built in statistical module.

#### 3.4.3. Processing DIA Raw Data with Focus on Selected Proteins

To verify the expression of selected proteins among the analyzed human carotid endarterectomies, we processed the collected DIA raw data files using Scaffold DIA (3.2.1). The first step of MS data processing involved converting the data files to mzML format using ProteoWizard (3.0.19254). For the spectral library search, runs were aligned based on retention times and individually searched against Homo_sapiens_prosit_generated_library (2).dlib with a peptide mass tolerance of 20 ppm and a fragment mass tolerance of 10.0 ppm. Carbamidomethylation of cysteine was considered a fixed modification. The digestion enzyme was trypsin with a maximum of one missed cleavage site(s) allowed. Only peptides with charges of 2 to 5 and a length of 6 to 30 were considered. Peptides identified in each sample were filtered by Percolator (3.01) to achieve a maximum FDR of 0.01 (1%). Individual search results were combined, and peptide identifications were assigned posterior error probabilities and filtered to an FDR threshold of 0.01 by Percolator (3.01). Peptide quantification was performed by Encyclopedia (1.12.31) [108]. For each peptide, the five highest-quality fragment ions were selected for quantitation.

### 3.5. Bioinformatics

Proteins that showed statistically significant differences in their expressions in the A and C samples compared to the H control carotid endarterectomies were submitted to Ingenuity Pathway Analysis^®^ (IPA^®^, QIAGEN, Redwood City, CA, USA) to derive bioinformatics annotations along with potential protein interaction networks, as well as associated biological functions and processes. Overlaps of p-values were reported from IPA^®^’s calculations using the right-tailed Fisher’s exact test [109]. The z-scores were generated for regulated functions, together with their predicted signaling patterns, through the MAP tool built into IPA^®^.

## Figures and Tables

**Table 1:** Summary of results from processing DDA raw data files by MaxQuant with MaxLFQ *.

Comparison	Statistical Analysis	Number of Differentially Expressed Proteins	Upregulated	Downregulated
A versus H	*t*-test	28	7	31
C versus H	*t*-test	79	25	54
C versus A	*t*-test	59	23	36

* Swiss-Prot *Homo sapiens* database; total number of identified proteins across all runs at 1% false discovery rate (FDR): 485; statistically significant differences in protein expression (*p* < 0.05, *n* = 5/group) were obtained from precursor-ion-intensity-based MaxLFQ quantification [34] through LFQ-Analyst [35]; fold change threshold: ≥2.

**Table 2:** Summary of results from searching DDA raw data files by Andromeda (MaxQuant’s engine) followed by validation and LFQ through Scaffold *.

Comparison	Statistical Analysis	Number of Differentially Expressed Proteins	Upregulated	Downregulated
A versus H	*t*-test	118	57	61
C versus H	*t*-test	317	95	222
C versus A	*t*-test	261	81	180
	One-way ANOVA	331		

* UniprotKB *Homo sapiens* database; total number of identified proteins across all runs (1% FDR, validation by MSFragger, Peptide Prophet, and Protein Prophet): 2740; statistically significant differences in protein expression (*p* < 0.05, *n* = 5/group) were assessed by spectral counting; fold change threshold: ≥2.

**Table 3:** Summary of results from processing DIA raw data files by Spectronaut *.

Comparison	Statistical Analysis	Number of Differentially Expressed Proteins	Upregulated	Downregulated
A versus H	*t*-test	184	42	142
C versus H	*t*-test	280	140	140
C versus A	*t*-test	213	124	89
	One-way ANOVA	709		

* UniprotKB *Homo sapiens* database; the total number of identified proteins across all runs (1% FDR): 3915 (by MSstatsShiny [42]); statistically significant differences in protein expression (*p* < 0.05, *n* = 5/group) were obtained by precursor-ion-intensity-based LFQ built into Spectronaut; fold change threshold: ≥2.

## Data Availability

The mass spectrometry proteomics data have been deposited to the ProteomeXchange Consortium via the PRIDE [110] partner repository with the dataset identifier PXD038922 and PXD056909.

## Supplementary Materials

The following supporting information can be downloaded at: https://www.mdpi.com/article/10.3390/ijms252413665/s1.
