# Supplementary material for: Identification of Protein Networks and Biological Pathways Driving the Progression of Atherosclerosis in Human Carotid Arteries Through Mass Spectrometry-Based Proteomics
Source: Int J Mol Sci. 2024 Dec 20;25(24):13665. doi: 10.3390/ijms252413665 (PMC11728284; doi:10.3390/ijms252413665)
Supplement: Supplementary file 1 [file ijms-25-13665-s001.zip › ijms-3354340_Fig S1.pdf]

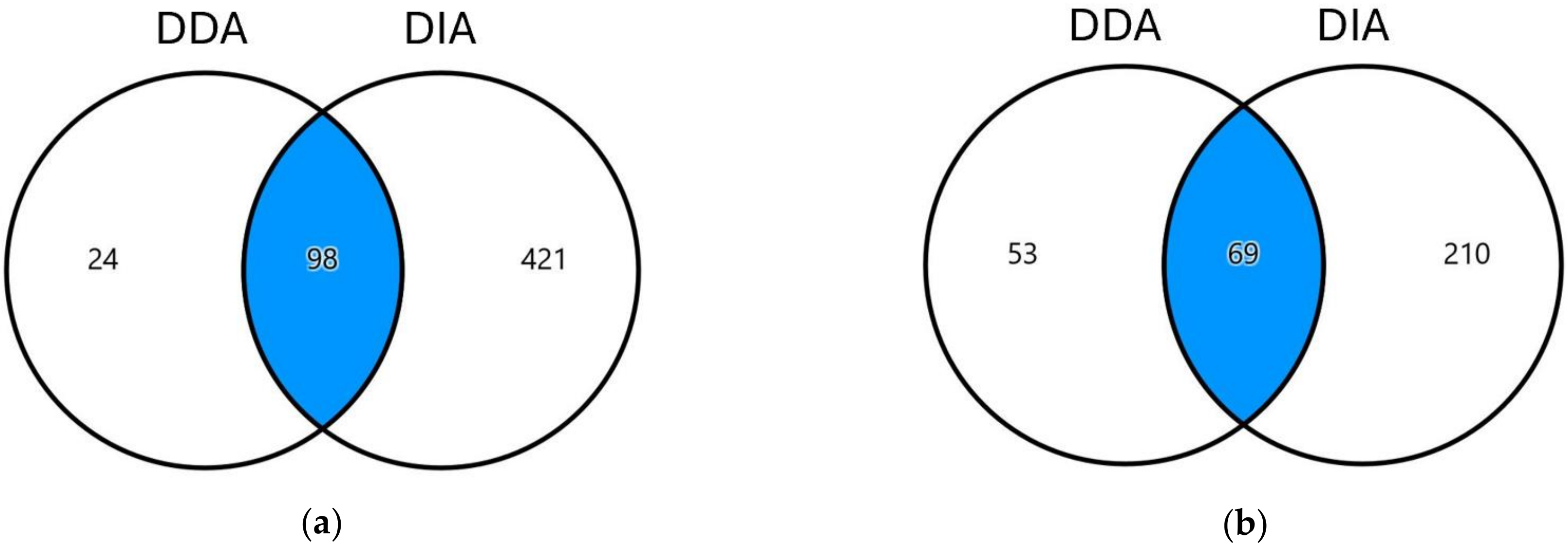

Figure S1. Venn diagram showing the overlap of DDA and DIA results based on proteins mapped by IPA®: (**a**) Number of proteins with statistically significant difference in expression by ANOVA ($p < 0.05$; fold change ≥ 2), (**b**) Number of proteins with statistically significant difference in expression upon pairwise group comparisons (unpaired *t*-tests, $p < 0.05$; fold change ≥ 2)
