# Supplementary figures and images for: Identification of Protein Networks and Biological Pathways Driving the Progression of Atherosclerosis in Human Carotid Arteries Through Mass Spectrometry-Based Proteomics
Source: Int J Mol Sci. 2024 Dec 20;25(24):13665. doi: 10.3390/ijms252413665 (PMC11728284; doi:10.3390/ijms252413665)

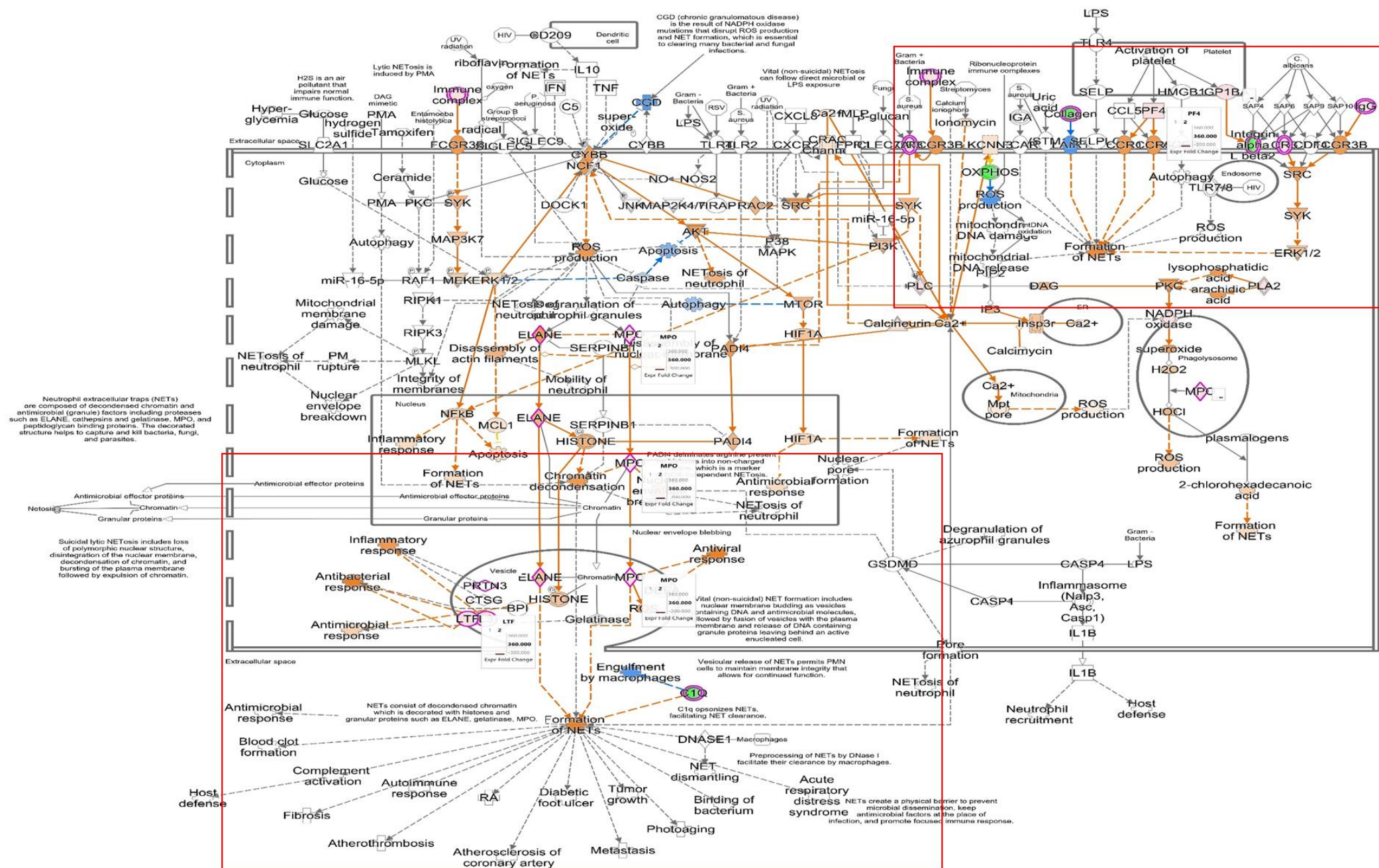

Supplement: Supplementary file 1 [file ijms-25-13665-s001.zip › ijms-3354340_Fig S2.pdf]
